# Postmenopausal Iron Overload Exacerbated Bone Loss by Promoting the Degradation of Type I Collagen

**DOI:** 10.1155/2017/1345193

**Published:** 2017-05-23

**Authors:** Qian Cheng, Xiaofei Zhang, Jun Jiang, Guoyang Zhao, Yin Wang, Youjia Xu, Ximing Xu, Haile Ma

**Affiliations:** ^1^The Second Affiliated Hospital of Soochow University, No. 1055 Sanxiang Road, Suzhou, Jiangsu 215004, China; ^2^Affiliated Hospital of Jiangsu University, No. 438 Jiefang Road, Zhenjiang, Jiangsu 212001, China; ^3^School of Medicine, Fudan University, No. 220 Handan Road, Shanghai 200433, China; ^4^School of Pharmacy, Jiangsu University, No. 301 Xuefu Road, Zhenjiang, Jiangsu 212013, China; ^5^School of Food and Biological Engineering, Jiangsu University, No. 301 Xuefu Road, Zhenjiang, Jiangsu 212013, China

## Abstract

117 postmenopausal women were divided into Normal, Bone loss (BL), and Osteoporosis group. Compared with Normal group (120.96 ± 43.18 *μ*g/L), the serum ferritin (Fer) in BL (223.37 ± 130.27 *μ*g/L) and Osteoporosis group (307.50 ± 161.48 *μ*g/L) was significantly increased (*p* < 0.05). Fer level was negatively correlated with BMD (*p* < 0.01). TRACP levels in Osteoporosis group (4.37 ± 1.69 U/L) were significantly higher than Normal group (4.10 ± 1.60 U/L,* p* < 0.05). ALP levels in Osteoporosis group (112.06 ± 62.05 U/L) were significantly upregulated compared with Normal group (80.22 ± 14.94 U/L,* p* < 0.05). *β*-CTX and PINP were the degradation products of type I collagen. *β*-CTX levels in Osteoporosis group (667.90 ± 316.55 ng/L) were significantly increased compared with Normal group (406.06 ± 112.12 ng/L,* p* < 0.05). PINP levels in Osteoporosis group (78.03 ± 37.31 *μ*g/L) were significantly higher than Normal group (37.60 ± 13.17 *μ*g/L,* p* < 0.01). More importantly, there was a positive correlation between serum Fer and PINP (*p* < 0.01). Serum Fer showed a positive correlation of serum *β*-CTX (*p* < 0.01). The overloaded iron improved the degradation of type I collagen.

## 1. Introduction

Iron was an essential trace element involved in human physiological functions [1, 2]. Iron deficiency can lead to bone loss and then induce osteoporosis [3, 4]. However, iron overload in the body had the toxic effect on osteoblasts [5], gave rise to osteoporosis by inhibiting osteoblast proliferation and differentiation, and increased osteoclastogenesis [6]. Overloaded iron aggravated the effects of ovariectomy on bone mass [7]. Iron overload in mice associated with elevated ferritin (Fer) and decreased Runx2 mRNA levels and inhibited osteogenic differentiation of mesenchymal stem cells [8, 9]. The zebrafish model of iron overload demonstrated an apparent inhibition of bone formation, accompanied by decreased osteoblast-specific genes expression [10]. 

Postmenopausal osteoporosis (PMOP) was a systemic bone metabolism disease, characterized by progressive bone loss following menopause [11]. Estrogen deficiency as a result of menopause was known to increase bone resorption and accelerate bone loss. Previous studies provided evidence that iron affected the bone mass only in the absence of estrogen, and the inhibition of estrogen on iron-induced osteopenia was particularly relevant to bone resorption rather than bone formation [7]. At present, iron overload is an important risk factor for PMOP [12].

Bone markers in serum can be determined for diagnostic and monitoring purposes for PMOP patients. Serum was recommended as test material over urine for reasons of better practicability and lower inter- and intraindividual variability [8]. Alkaline phosphatase (ALP) was secreted by the osteoblasts and promoted bone mineralization. Increased serum ALP was considered as a sign of primarily increased osteoblasts activity or secondarily as a corrective reaction of increased bone resorption [13]. Tartrate resistant acid phosphatase, type 5b (TRACP), was released into the bloodstream during bone resorption as bone-specific isoenzyme and generally reflected the activity of the osteoclasts and the extent of bone resorption [14].

Bone was composed of 17–20 wt.% collagen [15] and other components. The collagen of bone was considered to contribute to the toughness (energy to fracture) of the bone, mitigating the brittleness of the mineral, and contributed to bone strength [16]. Type I collagen as the most ubiquitous collagen constitutes approximately 30% of all proteins in extracellular matrix secreted by osteoblasts [17]. In addition, altered components and posttranslational modifications of collagen in extracellular matrix initiated and drove disease progression [18]. Type I collagen could be assessed as biomarkers for the synthesis of the bone matrix by measuring N-terminal propeptides of type I procollagen (PINP) and carboxyterminally cross-linked telopeptides (CTX) [19]. PINP occurred in the serum in two forms: as intact, trimeric peptide corresponding to the native separation product of procollagen during the synthesis of type I collagen and as monomeric peptide which was rather a degradation product of procollagen [20]. Type I collagen releases CTX into the bloodstream during bone resorption [21] including *α*-CTX and isomerized *β*-CTX form. However, *α*-CTX can only be determined in the urine [22].

In this study of a cohort of postmenopausal women, we evaluated the effect of iron overload on baseline characteristics (including age, years of menopause, Ca, P, BMI, liver and kidney function, glucose metabolism, lipid metabolism, and inflammatory response), BMD, TRACP, ALP, and type I collagen degradation products. We found that the association between iron overload and bone loss possibly originated from its role in promoting the degradation of type I collagen and then exacerbated the process of bone loss, induced osteoporosis, and fractures easily.

## 2. Materials and Methods

### 2.1. Patients and Bone Density Detection

One hundred and seventeen women in spontaneous menopause who came to physical examination center to perform a bone densitometry were enrolled in the study. Subjects with secondary osteoporosis, intestinal malabsorption diseases, or other kinds of deficient nutritional status were excluded.

Osteoporosis was determined according to the WHO criteria [23]. The study was approved by the human study review board of Jiangsu University in Zhenjiang and all the subjects signed an informed consent statement prior to their recruitment.

BMD was measured by a DPX-NT (Lunar Inc.) dual energy X-ray Absorption Spectrometry at lumbar spine and femoral neck according to the women clinical feature. The BMD unit was g/cm^2^ and was converted to a *T*-Score by the instrument. The diagnostic criteria for osteoporosis were according to WHO recommendations, *T* ≥ −1 (Normal), −2.5 < *T* < −1 (Bone Loss or Osteopenia), and *T* ≤ −2.5 (Osteoporosis). The instrument was calibrated before detection. Height and weight were registered and BMI was calculated.

### 2.2. Detection of Serum Iron, Baseline Characteristics, Bone Resorption, and Type I Collagen Degradation

All the patients measured baseline serum levels of blood calcium (Ca) and phosphorus (P), 25-hydroxyvitamin D (25-OH), hemoglobin, C-reactive protein (CRP), white blood cell count (WBC), creatinine, uric acid, total nitrogen and carbon dioxide, alanine aminotransferase (ALT), aspartate aminotransferase (AST), and glucose. Blood was drawn from an antecubital vein after an overnight fast of 10 or more hours and all the measurements were done from a single blood sample at a single time point per patient.

Serum Fer and TRF were assayed by electrochemical luminescence detection (Cobas 600, POCHE, Switzerland). Blood routine was detected by electrical impedance method (XN-3000, SYSMEX, Japan). Blood biochemical electrolytes were measured by enzymatic kinetic method (AU 5400, OLYMPUS, Japan). *β*-CTX and PINP were also evaluated with standard ELISA methods (Elecsys 2010, ROCHE, Switzerland).

### 2.3. Statistics

Three groups were initially formed by *T*-Score as Normal, Bone Loss, and Osteoporosis. In order to ensure the rationality of the grouping and the accuracy of subsequent data, principal component analysis (PCA, SPSS 19.0) was used to distinguish the three groups. The factors taken into PCA were age, Fer, TRF, 25-OH, *T*-Score of femoral neck and L_1_–L_4_, ALP, TRACP, *β*-CTX, PINP, BMI, Ca, P, years of menopause, hemoglobin, CRP, WBC, total N and CO_2_, creatinine, uric acid, triglycerides, total cholesterol, AST, ALT, and glucose.

Dunnett's Multiple Comparison Test (GraphPad Prism 5.0) was employed to compare the significance of bone resorption related biomarkers, Normal versus Osteoporosis or Normal versus Bone Loss group. The regression analysis (PCA, SPSS 19.0) was used for the correlation of serum iron and baseline characteristics, bone resorption, and type I collagen degradation.

## 3. Results

### 3.1. Verification of Experimental Grouping by PCA

**Figure 1:**
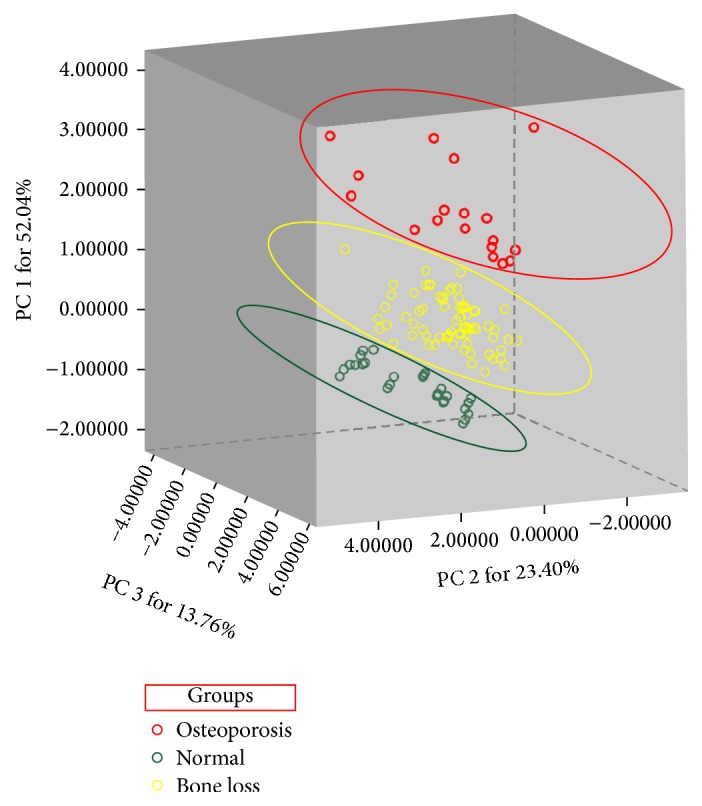
Verification of grouping by principal component analysis. First, we grouped according to the *T*-value of the femur and lumbar vertebrae (Normal *T* ≥ −1, Bone Loss −2.5 < *T* < −1, Osteoporosis *T* ≤ −2.5). Then, the PCA was performed on the data for further verification. Finally, the sample grouping scheme was determined on the basis of the results of the combined PCA and BMD. BMD and PCA were consistent with each other.

Grouped according to *T*-Score of femoral neck and lumbar spine (L_1_–L_4_), all the subjects were divided into Normal (*n* = 28), Bone Loss (*n* = 71), and Osteoporosis (*n* = 18) groups. In order to confirm that our grouping was statistically significant, in the PCA process, our operating procedures were as follows: date, date reduction, and factor analysis (the 26 variables mentioned in “Statistics” were calculated and regrouped by the PCA after being reduced into three principal components), and then their 3D scatter plot was obtained; one point represented a patient (Figure 1). The three principal components (PC1, PC2, and PC3) with >89% of the whole variance were extracted for analysis. PC1, PC2, and PC3 accounted for 52.04%, 23.40%, and 13.76% of the total variance, respectively. The remaining principal components had only a minor effect on the model and were discarded.

The dots of 117 patients were classified into three groups, Normal, Bone Loss, and Osteoporosis. This result was consistent with the *T*-Score group, indicating that the grouping of our experimental procedure was accurate and statistically significant.

### 3.2. Baseline Characteristics

In our cohort 71 women were affected by postmenopausal osteopenia and 18 women were affected by PMOP and 28 women were generally healthy. Patients and controls were not significantly different with regard to their baseline characteristics (Table 1), such as 25-OH, Ca, P, hemoglobin, WBC, total N and CO_2_, creatinine, and uric acid.

However, compared with Normal group, the age, years of menopause, BMI, and CRP of Osteoporosis group were significant differences (*p* < 0.05). The risk of osteoporosis was also likely to increase significantly as age and menopausal years increase. The BMI from Normal to Osteoporosis group showed a significant decrease trend (*p* < 0.05). Some studies had shown that obesity or excessive BMI was a risk factor for osteoporosis. It was found that the osteoporosis process was associated with a significant reduction in BMI.

**Figure 2:**
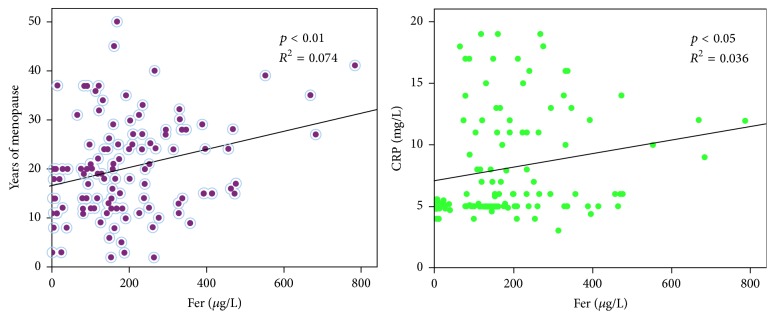
Correlative analysis of serum iron on years of menopause and CRP. Fer was positively correlated with years of menopause (*p* < 0.01). Fer was positively correlated with CRP (*p* < 0.05). The longer the menopause, the more serious the accumulation of iron, and the inflammatory response is promoted.

The increase in menopausal years accompanied by an increase in iron overload and a positive correlation appeared (*p* < 0.01). Serum iron was also positively correlated with the level of CRP (*p* < 0.01). The detailed results were shown in Figure 2.

### 3.3. Serum Iron and Bone Metabolism Biomarkers

**Figure 3:**
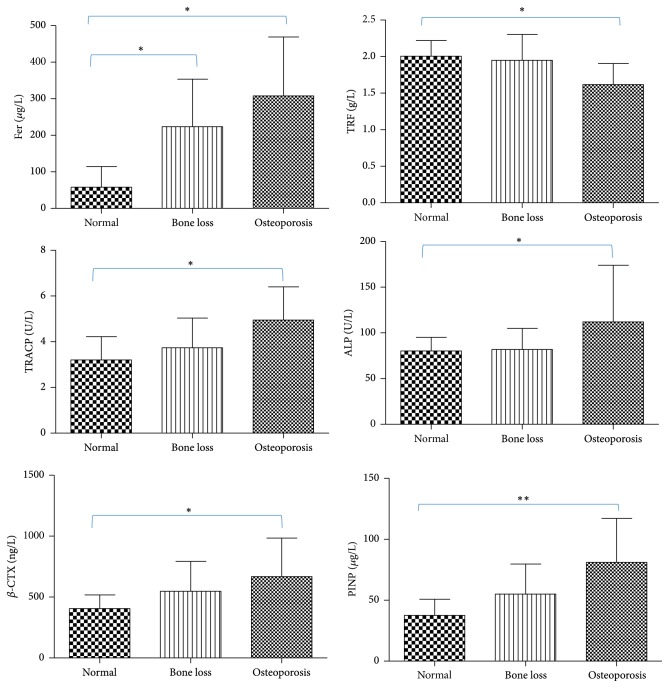
Values of serum iron, TRACP, ALP, *β*-CTX, and PINP in different groups. Compared with the Normal group, the Fer of the Bone Loss group and Osteoporosis group increased significantly (^*∗*^*p* < 0.05); the TRF of the Osteoporosis group decreased significantly (^*∗*^*p* < 0.05). TRACP, ALP, *β*-CTX, and PINP in Osteoporosis group were significantly upregulated by contrast with Normal group (^*∗*^*p* < 0.05 or ^*∗∗*^*p* < 0.01). PMOP was associated with iron overload and enhanced bone resorption.

The values of Fer, TRF, ALP, TRACP, *β*-CTX, and PINP were listed in Figure 3. The serum Fer levels in Normal, Bone Loss, and Osteoporosis were, respectively, 120.96 ± 43.18, 223.37 ± 130.27, and 307.50 ± 161.48 *μ*g/L. Compared with Normal, the other two groups were significantly increased (*p* < 0.05). The serum TRF levels in Normal, Bone Loss, and Osteoporosis group were, respectively, 2.00 ± 0.22, 1.95 ± 0.36, and 1.62 ± 0.29 g/L. The serum TRF levels in patients with osteoporosis were significantly lower than Normal group (*p* < 0.05).

Serum TRACP levels in Osteoporosis group (4.37 ± 1.69 U/L) were significantly higher than Normal group (4.10 ± 1.60 U/L, *p* < 0.05). Serum ALP levels in Osteoporosis group (112.06 ± 62.05 U/L) were significantly upregulated compared with Normal group (80.22 ± 14.94 U/L, *p* < 0.05). Serum *β*-CTX levels in Osteoporosis group (667.90 ± 316.55 ng/L) were significantly increased compared with Normal group (406.06 ± 112.12 ng/L, *p* < 0.05). Serum PINP levels in Osteoporosis group (78.03 ± 37.31 *μ*g/L) were significantly higher than Normal group (37.60 ± 13.17 *μ*g/L, *p* < 0.01).

### 3.4. Correlation between Fer and Type I Collagen Degradation

**Figure 4:**
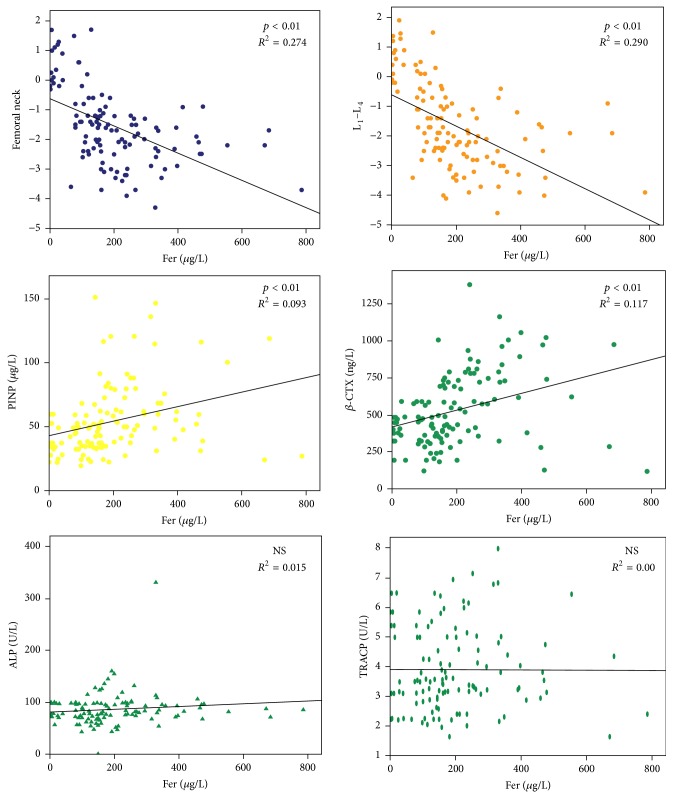
Correlation between serum iron and degradation of type I collagen. There was no correlation between serum iron, ALP, and TRACP. Elevated ALP and TRACP were not caused by iron overload. However, PINP and *β*-CTX levels were both positively correlated with iron levels (*p* < 0.01). Iron overload promoted the degradation of type I collagen.

Fer was not associated with ALP and TRACP, because Fer had no significant effect on serum ALP and TRACP levels. The serum Fer level, BMD, PINP, and *β*-CTX derived from 117 samples were taken into the correlation analysis; the result was shown in Figure 4. It was found that serum Fer level was significant negatively correlated with BMD (femoral neck and lumbar spine, *p* < 0.01). In addition, there was a significant positive correlation between serum Fer levels and serum PINP levels (*p* < 0.01). Serum Fer levels showed a significant positive correlation of serum *β*-CTX levels (*p* < 0.01).

### 3.5. Mechanism of Iron Overload on Type I Collagen Degradation

**Figure 5:**
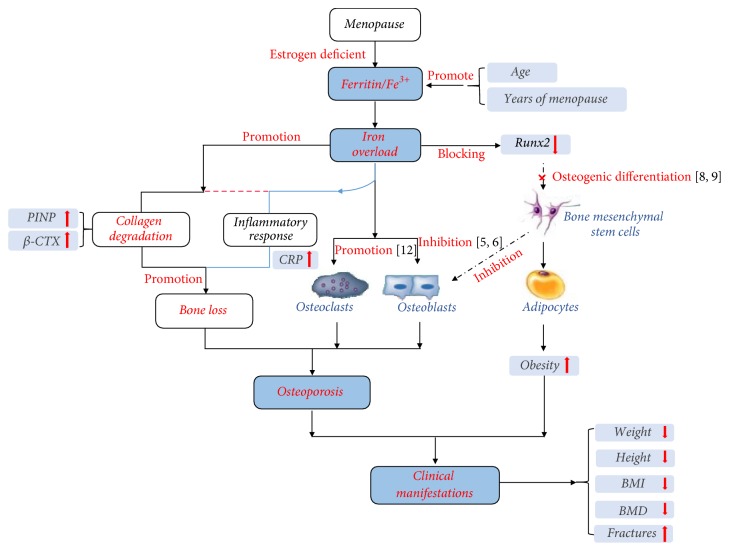
Mechanism of iron overload on PMOP. The overloaded iron improved the degradation of type I collagen and induced inflammatory response. Final performance was height reduction, weight loss, and BMI drop, and BMD decreased very significantly and caused osteoporosis.

According to our clinical data analysis, serum iron was significantly accumulated and then overloaded with the increase of menopausal years. The overloaded iron improved the degradation of type I collagen and induced inflammatory response in vivo significantly. All these processes performance for height reduction, weight loss, BMI drop, and finally BMD decreased and caused osteoporosis. This mechanism was shown in Figure 5.

## 4. Discussion

BMD was the most important indicator in clinical diagnosis of osteoporosis [24]. However, only grouping based on *T*-values of BMD (femoral neck and lumbar spine) did not describe the overall information of the samples. If the samples were grouped incorrectly, then the comparison between groups was not scientific and the experimental results may be errors. PCA was a multivariate data analysis and could be performed to define both similarities and differences among complex samples [25–27]. Therefore, we used the PCA for group validation after the BMD grouping; if the two results were inconsistent, we had to readjust the sample grouping. Fortunately, PCA results further confirmed the rationality and feasibility of three groups according to *T*-values. We observed that 117 sample dots obtained from 26 variables were successfully classified into Normal, Bone Loss, and Osteoporosis group with no overlap between each other. Thus, our follow-up targeted comparison of the indicators between groups was statistically significant.

The menopause age of Chinse women was generally about 50 years [28]; often the older the case, the more the years of menopause and the lower the estrogen level in the body. Therefore, the average age of women with osteoporosis was larger than normal. As the postmenopausal age increased, their bone mass decreased gradually from normal to osteopenia to osteoporosis. The height and weight of osteoporosis patients both showed significant downward trend and ultimately caused their BMI index to be significantly lower. CRP was an important indicator of inflammatory response. The CRP in Bone Loss and Osteoporosis group was significantly increased which demonstrated that osteopenia or osteoporosis was associated with an inflammatory response [29]. Therefore, study of the inflammatory pathways or related pathways of osteoporosis was really an important method for treatment of osteoporosis.

Lots of in vitro studies showed that iron salts could inhibit the growth of hydroxyapatite crystals in an acellular and nonenzymatic model of calcification which suggested a direct inhibitory effect of iron on bone mineralization [30], independent of cells, proteins, and enzymes. So the serum Fer in patients with osteoporosis significantly increased with the BMD significant decrease. This reaffirmed the negative effect of iron on BMD, and the more the menopausal years, the more serious the iron overload. Besides, serum iron accumulation may also be one of the important factors leading to inflammatory response in patients with osteoporosis, as CRP increased with the rise of Fer.

*β*-CTX and PINP were the degradation products of type I collagen [31]. Serum Fer levels had a positive effect on both *β*-CTX and PINP. Furthermore, in-depth analysis showed that iron overload reduced bone density by promoting the degradation of type I collagen. The effect of iron overload on collagen degradation was the first reported by us. Subsequently, through the follow-up literature search from PubMed, it was found that metal ions did promote or inhibit the degradation of collagen; for example, Osorio et al. found that zinc in excess reduced matrix metalloproteinases—mediated collagen degradation [32].

In our research, there are still some deficiencies; for example, the mechanism of iron overload on the degradation of type I collagen is unclear. Our follow-up study will investigate the regulatory enzymes of collagen degradation and the interaction of serum iron and the specific regulatory enzymes.

## 5. Conclusions

The overloaded iron improved the degradation of type I collagen and is accompanied by bone toughness reduction. Promoting collagen degradation may aggregate the process of bone loss under estrogen-deficient environment. It is an innovative discovery of PMOP pathogenesis.

## Figures and Tables

**Table 1:** Baseline characteristics in Normal, Bone Loss, and Osteoporosis groups series.

	Patients		
	Normal	Bone Loss	Osteoporosis
	(28)	(71)	(18)
Ages (yrs)	66.89 ± 11.54	70.61 ± 9.11^▲^	77.61 ± 10.43^*∗*^
Femoral neck (*T*-Score)	−0.39 ± 0.88	−1.75 ± 0.75	−3.22 ± 0.53
L_1_–L_4_ (*T*-Score)	−0.36 ± 0.62	−1.87 ± 0.88	−3.53 ± 0.53
25-OH (mmol/L)	43.59 ± 9.13	43.05 ± 19.36	44.07 ± 22.71
Height (cm)	158.33 ± 5.89	158.55 ± 3.71	154.94 ± 4.48
Weight (kg)	57.89 ± 9.43	59.03 ± 8.96	49.94 ± 9.61
BMI	23.12 ± 3.76	23.38 ± 3.10	20.74 ± 3.61^*∗*^
Ca (mmol/L)	2.25 ± 0.10	2.24 ± 0.14	2.18 ± 0.13
P (mmol/L)	1.06 ± 0.18	1.10 ± 0.19	1.11 ± 0.24
Years of menopause (yrs)	15.89 ± 9.71	19.99 ± 8.63	27.33 ± 11.79^*∗∗*^
Hemoglobin (g/L)	113.33 ± 16.05	117.66 ± 19.73	105.11 ± 16.02
CRP (mg/L)	8.47 ± 4.37	8.77 ± 4.56^▲^	9.74 ± 4.98^*∗*^
WBC (10^9^/L)	6.92 ± 2.00	6.80 ± 2.16	6.83 ± 1.51
N (%)	70.56 ± 10.55	69.66 ± 9.84	71.35 ± 9.29
Total CO_2_ (mmol/L)	26.66 ± 2.21	25.27 ± 2.20	25.22 ± 6.01
Creatinine (U/L)	53.22 ± 8.47	56.45 ± 20.05	58.06 ± 13.14
Uric acid (*μ*mol/L)	213.56 ± 36.37	268.35 ± 74.62	232.33 ± 79.38
Triglycerides (mmol/L)	0.87 ± 0.33	1.25 ± 0.71	1.33 ± 1.52
Total cholesterol (mmol/L)	4.54 ± 1.14	4.91 ± 1.02	4.67 ± 0.97
AST (U/L)	21.00 ± 5.59	25.34 ± 19.13	24.56 ± 11.42
ALT (U/L)	17.56 ± 7.76	20.03 ± 20.88	19.28 ± 12.82
Glucose (mmol/L)	6.28 ± 0.89	5.71 ± 1.06	5.83 ± 0.98

*Note*. ▲ means significant difference compared with the Normal group, *p* < 0.05. *∗* means significant difference compared with the Normal group, *p* < 0.05. *∗∗* means significant difference compared with the Normal group, *p* < 0.01.
